# The role of intervention mapping in designing disease prevention interventions: A systematic review of the literature

**DOI:** 10.1371/journal.pone.0174438

**Published:** 2017-03-30

**Authors:** Rayyan M. Garba, Muktar A. Gadanya

**Affiliations:** 1 Department of Community Medicine, Aminu Kano Teaching Hospital, Kano, Nigeria; 2 Department of Community Medicine, Bayero University/ Aminu Kano Teaching Hospital, Kano, Nigeria; The Hospital for Sick Children, CANADA

## Abstract

**Objective:**

To assess the role of Intervention Mapping (IM) in designing disease prevention interventions worldwide.

**Methods:**

Systematic search and review of the relevant literature—peer-reviewed and grey—was conducted using the Preferred Reporting Items for Systematic Reviews and Meta-analysis (PRISMA) guidelines.

**Findings:**

Only five of the twenty two included studies reviewed were RCTs that compared intervention using IM protocol with placebo intervention, and provided the outcomes in terms of percentage increase in the uptake of disease-prevention programmes, and only one of the five studies provided an effect measure in the form of relative risk (RR = 1.59, 95% CI = 1.08–2.34, p = 0.02). Of the five RCTs, three were rated as strong evidences, one as a medium evidence and one as a weak evidence, and they all reported statistically significant difference between the two study groups, with disease prevention interventions that have used the intervention mapping approach generally reported significant increases in the uptake of disease-prevention interventions, ranging from 9% to 28.5% (0.0001 ≤ p ≤ 0.02), On the other hand, all the 22 studies have successfully identified the determinants of the uptake of disease prevention interventions that is essential to the success of disease prevention programmes.

**Conclusion:**

Intervention Mapping has been successfully used to plan, implement and evaluate interventions that showed significant increase in uptake of disease prevention programmes. This study has provided a good understanding of the role of intervention mapping in designing disease prevention interventions, and a good foundation upon which subsequent reviews can be guided.

## Introduction

### Background

Health promotion entails the use of both educational and environmental interventions to improve conditions of living favourable to health.[1] Different health promotion intervention models have been used in the past, such as the logic model described by Kirby and associates[2] and the PRECEDE/PROCEED model described by Green and Kreuter.[1] The logic model provides a background of risk behaviours and their determinants in the at-risk groups. It also depicts the environmental factors and their determinants that impact directly or indirectly on the risk behaviours, as well as identifying characteristics that distinguish between effective and ineffective programmes.[2] The PRECEDE component involves an analysis of the behavioural and environmental determinants of health and their correlates. While the PROCEED component involves the development, implementation and evaluation of a health promotion programme.[1] Intervention mapping (IM) is a health promotion protocol for selecting and applying social and behavioural science theories, such as theories of health psychology, to the planning, implementation and evaluation of health promotion programmes.[3]

Intervention mapping is not a new health promotion theory, it is a framework that tries to bridge the gap between theories and practice, because despite the wide range of social and behavioural science theories, their use in the development and implementation of health promotion programmes has been a constraint to planners.[4] According to Kok and Mesters, the concept of intervention mapping, came into practice because of the problems encountered from using existing models such as:[5]

“*trying to change behaviour that was not related to the problem*, *trying to change determinants for behaviours that were not relevant to the behaviour*, *trying to change individual behaviour while environmental factors were responsible*, *trying to apply change methods that were never shown to be effective*, *trying to implement programmes by health professionals that were inadequately trained to do so*, *and so forth”*.

These led to a retrospective review of prototype health promotion programmes to come up with the intervention mapping protocol which describes a health promotion intervention in six steps as shown in Table 1. Although the process is defined by a series of steps, the planning process is iterative and cumulative rather than linear, with programmers moving in both directions as new themes and concepts are evolving, and each step depends on findings of the preceding step.[3]

**Table 1:** Intervention mapping steps, adapted from Bartholomew et al.[3].

Intervention mapping steps	Tasks
**One: Needs Assessment**	Plan for needs assessment Assess health status, quality of life, behaviour, environment and capacity Define programme outcomes.
**Two: Definition of proximal programme objective matrices**	Clarify expected changes in behaviour and/or environment Define performance objectives Define correlates of the target behaviour change of the population at risk Match correlates to performance objectives to produce matrices of proximal programme objectives.
**Three: Selection of theory-based methods and practical strategy**	Review programme with intended participants Identify relevant theoretical methods Select programme methods Select or design strategies Match strategies to performance objectives
**Four: Production of programme components and design**	Consult with all stakeholders Design the scope, sequence, theme and resource list of the programme Review available resources Develop materials of the programme Pre-test the materials with all target stakeholder group
**Five: Programme adoption and implementation plan**	Identify adopters and users Define performance objectives for adoption, implementation and sustainability Match adoption and implementation performance objective with personal and external correlates to create matrix Select methods and strategies Design interventions to match programme
**Six: Evaluation plan**	Programme description Description of outcomes and effects of the programme Identify questions base on the matrix and process Identify indicators and their measures Specify designs and plan of evaluation

Step one above is based on the PRECEDE model described by Green and Kreuter[1] and is done before starting the actual intervention plan. It entails an assessment of the at risk population by reviewing all relevant literature related to the problem area, as well as collecting new information from the community though interviews and focus groups to have a better understanding of attitudes, beliefs and behaviours.[3] Step two forms the basis for the intervention by stating who and what is expected to change at both individual and ecological levels by stating the performance objectives and their determinants to produce the matrices of proximal performance objectives.[3] Each of the performance objectives has determinants, for example, self-efficacy is a determinant for choosing not to have sex and confidence is a determinant for negotiating condom use.[6] Step three entails the selection of theory informed methods and practical strategies. An intervention method is a process of using theory-based approach to change behaviour or environmental conditions,[3] for example, social learning theory (Bandura) has been used as the basis for methods improving self-efficacy and skills, theory of reasoned action (Fishbein & Ajzen) for changing subjective normative belief and behavioural beliefs, health belief model (Rosenstock) for changing knowledge, and risk perception.[6] Step four basically involves description of the programme protocols and contexts by participants, as well as pilot-testing the strategies and materials. It provides the vehicles for conveying the program design to producers.[3] In step five, programme sustainability is also considered in addition to adoption and implementation. In step six, the evaluation plan (which starts in step one, and developed together with the intervention map) is finalized.[3] This involves both process and effect evaluation. Process evaluation basically assess the fidelity of implementation, while effect evaluation assesses the impact of the intervention in the target population.[3] This can be done by randomizing intervention population with non-intervention populations to assess the effect of the intervention.[3,7]

The intervention mapping framework has been used to successfully adapt health promotion programmes.[6,8] When adapting a programme from one population to another using the intervention mapping protocol, each of the tasks of all the steps described in Table 1 must be considered in terms of the following: what is still relevant to and can be maintained in the new population, what needs to be added for the new population, what needs to be deleted as inappropriate for the new population and what needs to be deleted or adapted as impractical in the new population.[6] This is also achieved by reviewing all the relevant literature, collecting new data from the new population through individual interviews and focus groups, as well as key informant interviews with major stakeholders.[6]

Despite its widespread use in designing health promotion and disease prevention programmes, little evidence exists on the magnitude of the role IM plays in promoting uptake of disease prevention interventions. Therefore, this review aims at critically appraising the literature to assess the role of intervention mapping in designing disease prevention interventions worldwide.

## Methods

### Search strategy and selection criteria

The selection criteria was jointly decided and agreed by the two reviewers based on the study objective. This research is limited to studies that used intervention mapping in disease-specific prevention interventions, as shown in Table 2.

**Table 2:** Eligibility criteria.

	Inclusion criteria	Exclusion criteria
Language	Only studies published in English	Studies published in languages other than English
Date of publication	From 1999 to 2014	
Publication status	Peer reviewed journal articles and reviews, and grey literature (mainly unpublished research).	Systematic reviews, conference abstracts, editorials.
Type of data	Qualitative and quantitative	
Study design	Any study that uses only the intervention framework to design a disease prevention intervention	
Study population	All ages and both genders	
Intervention	Disease-specific prevention interventions that used the intervention mapping protocol	Non disease-specific interventions even if based on the intervention mapping protocol such as; IM studies on physical activity, healthy eating, sedentary lifestyle, overweight/ obesity, cigarette smoking, alcohol, adherence to treatment, quality improvements, studies that combined intervention mapping with other forms of interventions or modified the intervention mapping framework, and studies that used intervention mapping to adapt an existing programme to a new population, as they tend to measure mainly the success of programme adaptation

The PRISMA guidelines for reporting systematic reviews was followed to allow for systematic reporting. The PRISMA checklist is shown as File A in S1 Supporting Information.[9] Search for published literature was conducted in three main electronic databases: MEDLINE, EMBASE and Web of Science. It was initially designed using MEDLINE on 8th August 2014 and then adapted to the other databases on the same date using their specific terms. This allowed for testing of precision and sensitivity. The Campbell Collaboration and the Cochrane Library were also searched for any available and/or on-going systematic reviews on the topic. Search for grey literature (mainly unpublished research) was carried out on the following databases: OpenGrey and NYAM Grey Literature Report.

A very broad search was initially conducted, which aimed at identifying all intervention mapping studies on health promotion and disease prevention programmes. The titles and abstracts of all the identified studies were read to select those that used intervention mapping in disease-specific prevention interventions. After going through series of modifications, two main search categories were identified; “intervention mapping” related and “disease prevention” related. This gave the broadest search that captured all the relevant studies. The choice of disease prevention related terms was guided by a listing of areas of health promotion that intervention mapping has been applied, as stated on the intervention mapping website.[10] However, this underwent a series of iterative process leading to modifications as recommended in the PRISMA guidelines.[11] The Boolean operators AND/OR and truncation were used to link words and to identify all the possible endings of the search terms respectively as shown below:

Intervention mapping AND (HIV OR human immunodeficiency virus OR AIDS OR acquired immune deficiency syndrome OR hepatitis B virus OR HBV OR human papilloma virus OR HPV OR chlamydia OR influenza OR infect* OR injur* OR breast cancer OR cervical cancer OR prostate cancer OR colon cancer OR cancer OR drug* OR sex* OR smoking OR cigarette OR alcohol OR drinking OR binge OR wine OR bear OR behavi#r OR physical activity OR exercise OR sedentary OR inactivity OR psychiatr* OR psycholog* OR mental OR fruit OR vegetable OR diet OR nutrition* OR eat* OR feed* OR low calorie* OR low energy OR low fat OR low salt OR obes* OR weight gain OR overweight OR worker* OR student* OR depress* OR dementia OR stress OR chronic disease OR diabetes OR hypertension OR vaccin* OR stroke OR disabilit* OR asthma OR gynecolog* OR health promotion OR health education OR prevention OR ehealth OR family-based OR family OR school-based OR school OR web-based OR workplace OR work related).

Both indexed and free-text terms for intervention mapping were searched for. Details of the search strategy are attached as File B in S1 Supporting Information.

### Data extraction and quality of studies

All the identified studies were transferred to Endnote X7 and duplicates were removed. Ineligible studies were removed after reading the abstracts, while the full texts of the eligible studies were obtained and read for further ascertainment of eligibility or otherwise. Data extraction was carried out independently by the two authors, and compared for consistencies.

#### Critical appraisal

This involved an assessment of methodological quality as well as the risk of bias in the eligible studies. The appraisal status was not meant to ascertain eligibility status of the studies, but to identify strengths and weaknesses. Since the intervention mapping protocol designs and assesses the effect of an intervention, a combination of (mainly) the quality assessment tool for quantitative studies developed by the Effective Public Health Practice Project (EPHPP) and (to a lesser extent) the Centre for Evidence-Based management (CEBMa) checklist for surveys were considered appropriate, because the last step of the intervention mapping framework involves an evaluation of the intervention effect in quantitative terms. The EPHPP tool uses eight items to assess the quality of quantitative studies.[12] Each of these eight items is rated as strong, moderate or weak to collectively give rise to an overall methodological ratings of strong (four strong ratings with no weak ratings), moderate (less than four strong ratings and one weak rating) or weak (two or more weak ratings) evidence.[13] Details of the quality appraisal are attached as File C in S1 Supporting Information. Results of the critical appraisal were used to guide construction of the tool for data extraction and synthesisAuthors were contacted where information was not clear or when documents were not available.

## Results

### Literature search and study selection

**Fig 1:**
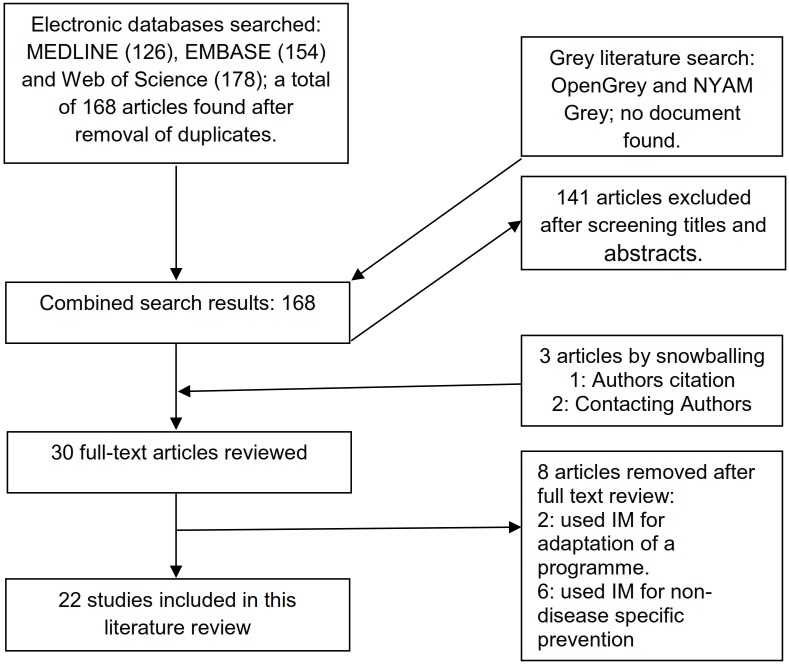
Flow chart for study selection.

Outcome of the literature search and selection of studies is summarized in Fig 1. The Cochrane library and the Campbell collaborations were searched for existing systematic reviews but none was found. Since most of the studies did not provide information on the effects of the interventions, authors were contacted to obtain that information. Out of the 16 authors contacted, only 6 responded at the time of compiling his review. Two authors provided the documents,[14,15] while the other four said the intervention effect trials were not yet published.[16–19]

### Study characteristics

A total of 22 studies were included in this review. Three studies were cluster RCTs,[20–22] and two are individual RCT.[23,24] Twelve studies were conducted in the Netherlands,[14,17,21,22,25–31] five were conducted in the U.S.A,[15,16,19,32,33] two were conducted in Taiwan,[23,34] one was conducted in Tanzania,[35] and one was conducted in both Tanzania and South Africa.[18]

Seven studies were on HIV/STI prevention,[18,19,25–28,35] one was on chlamydia prevention,[30] one was on Hepatitis B virus (HBV) prevention,[29] four were on influenza prevention,[17,21,22,31] four were on cervical cancer prevention,[15,16,23,34] one was on breast & cervical cancer prevention,[33] one was on secondary stroke prevention,[32] and one was on physical activity related injury prevention.[14]

Byrd et al (2013), Collard et al (2010), Looijmans-van den Akker et al (2010) and Hou et al (2002) were effect evaluation trials for Byrd et al (2012), Collard et al (2009), Looijmans-van den Akker et al (2011) and Hou et al (2004) respectively. Thus, they reported findings on different components of the same studies.

### Assessment of risk of bias

Since only five studies were RCTs out of which only one provided an effect estimate in form of relative risk, and also considering the high possibility of heterogeneity, the risk of publication bias could not be assessed using funnel plot. However, the possibility of selection bias, the study design, possibility of confounders, blinding, data collection methods, withdrawal rate, intervention integrity and robustness of analysis, as contained in the EPHPP tool, were assessed to rate the methodological qualities of the studies as described in Appendix 3.

### Heterogeneity

Even though all the studies used intervention mapping to design the respective disease prevention interventions, there is still a high possibility of heterogeneity (both clinical, methodological and statistical) because most of them differ in the following ways; epidemiologic design, sampling technique, study population, disease categories, methods of data collections and methods/robustness of data analysis. However, heterogeneity was not formally assessed because meta-regression could not be performed due to incomplete data, and sub-group analysis stands the danger of over interpretation.

### Results of individual studies and synthesis of results

A summary of study characteristics is presented in Table 3, and the summary of findings is provided afterwards, while the adapted appraisal tool is summarized in Table 4.

**Table 3:** Summary of results.

Studies (Author, date)	Intervention type	Study population & sampling	Application of the IM framework	Methods of data collection & analysis	Outcome and effect measure
Byrd et al, 2013[23]	Individual RCT on Pap-smear screening for Cervical cancer, the AMIGAS intervention.	Hispanic women of Mexican heritage aged 21 years and above, in the United States of America. 631 women were recruited based on in-person approach at shopping malls, schools, community centres, retail stores and churches.	IM was used to develop role modelling video, flip charts, games and hand outs. Details described in a different paper; Byrd et al, 2012	Data on the primary outcome (cervical cancer screening) was obtained by self-reporting in a follow up survey, validated through review of medical records. Computer generated randomization was done and data was analysed by both intent-to-treat and per-protocol methods, with the level of statistical significance set at 0.05	By intent-to-treat analyses, 52.3% of women in the intervention group reported taking up screening, while 23.8% in the control group reported screening uptake (p<0.0001). By per-protocol analysis, 61.7% in the intervention group and 28.6% in the control group reported screening uptake (p<0.0001)
Theunissen et al, 2013[30]	Chlamydia trachomatis screening	High-risk young people aged ≤ 25 years, who are partners of Chlamydia trachomatis positive young people of the same age in the Netherlands. Web-based respondent driven (chain referral) sampling was used for partner notification	Authors demonstrated good understanding and application of the IM framework in developing a theory based Chlamydia screening intervention, following the first five steps in detail, but not the sixth step	Semi structured interviews were conducted to obtain data on the needs assessment (8 women and 13 men). Participants received email and/or text message from their partners to login to the website where a questionnaire is filled and decision is taken whether (and how) to take up the screening test. Quantitative data analysis was not described in this article	Quantitative effect/outcome measures of the evaluation were not provided in the article
Riphagen-Dalhuisen et al, 2013.[21]	Cluster randomized control trial of Influenza vaccination for health care workers (HCWs) in acute settings over 2 influenza seasons	Health care workers in six Dutch University Medical Centres, in the Netherlands. All eligible participants in a cluster were sampled	Authors demonstrated good understanding and application of the IM framework in developing a theory based influenza vaccination intervention, following the first 5 steps in detail and 6th step to a lesser extent	Needs assessment data was obtained from a questionnaire-based study from 11 determinants associated with influenza vaccine compliance were obtained using a multivariable analysis, with odds ratios ranging from 1.7 to 28.9. Both qualitative and quantitative programme evaluation data were obtained using a web-based questionnaire in the following season, but detailed analysis not described	The effect in the intervention (IM) clusters relative to the control clusters was not given.
Byrd et al, 2012.[15]	Cervical cancer screening using pap-smear testing, the AMIGAS intervention	Hispanic women of Mexican heritage living in Texas-Mexican Border in the United States of America. Systematic random sampling of 10 households from each of the randomly selected 50 blocks groups was conducted	Authors demonstrated good understanding and application of the IM framework in developing a theory based Pap-smear screening intervention, following the first five steps in detail, but not the sixth step	13 focus groups and literature review were used to obtain data for needs assessment and quantitative surveys for the intervention. Both qualitative and quantitative data were obtained, but details of data analysis were not shown.	The intervention is being evaluated in a separate randomized controlled trial, hence effect estimates not provided in this paper
Scarinci et al, 2012.[16]	Cervical cancer prevention based on sexual risk reduction (Primary) and pap smear (secondary) testing.	Latina immigrant women in the United states of America. Door to door invitation approach was used to sample participants in all the identified sites	Authors demonstrated good understanding and application of the IM framework in developing a theory based cervical cancer prevention intervention, following all the six steps in details	Needs assessment data was obtained from focus groups and quantitative survey in the target population. Group randomization was used for programme evaluation, however, details of data collection and analysis for the intervention is not provided in this paper	The programme effect was not described in this paper.
Wolfers et al, 2012.[28]	Sexually transmitted infection (STI) testing, the ROsafe intervention	Vocational schools students in the Netherlands. Sampling method not described.	Authors demonstrated good understanding and application of the IM framework in developing a theory based STI testing intervention, describing the first four steps in detail, and steps 5 and 6 to a lesser extent	Needs assessment data were obtained from semi structured interviews (n = 38) and a quantitative survey (n = 778). With detail results and analysis presented. Data collection method for the evaluation was not presented in this paper.	Data and analysis on the intervention effect evaluation said to be provided in a separate randomized controlled trial
Van Der Veen et al, 2011.[29]	Hepatitis B Virus (HBV) screening	First generation Turkish immigrants aged 16–40 years in Rotterdam, the Netherlands. The sampling technique was not described	Authors demonstrated good understanding and application of the IM framework in developing a theory based HBV screening intervention, following all the six steps in details.	Focus groups and survey data were used for needs assessments. While a web-based questionnaire was used for the intervention data collection. Multivariate analysis was conducted for the survey to get the determinants of screening. But the effect evaluation analysis is not described in this paper	Taking a STI test is the main outcome measure, but evaluation of the intervention effect is been described in a separate randomized controlled trial
Looijmans-van den Akker et al, 2011.[31]	Influenza vaccination	Health care workers in nursing homes in the Netherlands. All nursing homes were (n = 335) were used for needs assessment, and 6636 HCWs were randomly sampled from the 36 nursing homes that agreed to participate in the intervention.	Authors demonstrated good understanding and application of the IM framework in developing a theory based influenza screening intervention, describing all the six steps in details.	In-depth interviews, focus groups and quantitative surveys were conducted to obtain data on needs assessment. A cluster randomized controlled trial was conducted to assess the effect of the intervention, but described in a separate paper.	A separate evaluation cluster randomized control trial (Looijmans-van den Akker et al, 2010) showed that the program intervention institutions had a 9% increase in the uptake of vaccination compared with the control institutions.
Kok et al, 2011.[17]	Influenza vaccination	Health care workers in hospitals and nursing homes in the Netherlands. Sampling technique not described	Authors demonstrated good understanding and application of the IM framework in developing a theory based influenza screening intervention, following all the six steps in details.	Details of data collection methods and analysis were not provided in this paper	Effect estimate was not provided in this paper.
Collard et al, 2010.[20]	Cluster RCT of the iPlay intervention on Physical activity (PA) injury prevention,	School children aged 10–12 years in the Netherlands. 40 schools (2210 students) were randomly selected and included in the study.	IM was used to design the iPlay intervention details of which is provided in another paper; Collard et al, 2009.	The primary outcome (number of injury per 1000 hours of sports participation, IID) was recorded by physical education teachers. Randomization (with schools as units) was based on computer generated random numbers. Intention-to-treat analysis was performed. Hazard ratio was estimated using multilevel Cox proportional hazard regression analysis, and the difference in injury severity was assed using multilevel logistic regression. P-values and 95% CI were provided.	The total PA injuries were 100 and 104 in the intervention and control groups with IIDs 0.38 (95% CI; 0.32–0.46) and 0.48 (95% CI; 0.38–0.57) respectively. However, the intervention resulted in a 50% reduction in IID in the low active (<414 minutes of PA per week) group (HR, 0.47; 95% CI, 0.21–1.06). Also a >50% reduction in sports and leisure time injuries (HR, 0.23; 95% CI, 0.07–0.75) and (HR, 0.43; 95% CI, 0.16–1.14)
Looijmans-van den Akker et al, 2010.[22]	A cluster randomized control trial evaluating the effect of a multi-faceted influenza vaccine program.	Health care workers (HCWs) in nursing homes in the Netherlands. 36 (11% of all) nursing homes that agreed to participate were randomly allocated to intervention and control groups by a computer programme, making 18 per group. 2 homes left the intervention group for personnel shortage and 1 home left the control group for no vaccination offered in the period, with a total of 3363 HCWs in all.	Details of IM application was described in a different paper (Looijmans-van den Akker et al, 2011)	Sample size calculation for cluster RCTs was applied with at least 12 clusters per group to detect a minimum increase of 10.5% to 25%. SPSS was used for data analysis using generalised estimation equation to take into account, the cluster design effect. Relative risk, 95% confidence interval and p-values were presented to all results.	The primary outcome is the proportion of HCWs that were vaccinated against influenza in both the intervention and control groups. The influenza vaccine uptake in the intervention group was 9% higher than the control group (RR = 1.59 95%CI = 1.08–2.34, p = 0.02)
Corbie-Smith et al, 2010.[19]	HIV prevention	African Americans in rural eastern North Carolina, United states of America. Sampling technique was not described.	Authors demonstrated good understanding and application of IM in developing a theory based HIV prevention intervention, but describing only the first four steps.	Need assessment data was obtained from focus groups and in-depth interviews. However, details of intervention data collection and analysis were not provided in this paper.	The behavioural outcomes identified were: abstinence, condom use among sexually active, and healthy dating/relationship. Effect evaluation of the programme outcomes after implementation was not provided in this paper.
Schmid et al, 2010.[32]	Secondary stroke prevention	People with history of stroke or transient ischemic attack in Indianapolis and Houston, United states of America. Sampling technique was not described.	Authors demonstrated good understanding and application of IM in developing a theory based secondary stroke prevention intervention describing all the steps.	Structured interviews were used to collect data for needs assessment. Other details of data collection and analysis were not provided in this paper.	Determinants of prevention were found to be the need for provider (discharge) check-off list, clinical reminders, training and education on risk factors and local resources, stroke support groups, IEC materials and administration support. Programme effect measure for the evaluation was not provided in this paper.
Collard et al, 2009.[14]	Physical activity related injury (PARI) prevention, the iPlay intervention	Primary school children in the Netherlands. 520 out of the 7000 primary schools were randomly selected from a database, and all children were eligible for inclusion in the study.	Authors demonstrated good understanding and application of IM in developing a theory based physical activity related injury prevention intervention, describing all the six steps in clear details.	Individual and focus groups interviews were conducted for needs assessment. Questionnaires were filled by students with PARI identified by the physical education (PE) teachers in a cluster RCT involving 500 children per group (intervention/control), aimed at getting a significant difference in the incidence (7%) of PARI at a power of 90%, 5% significance level and 10% intra cluster correlation coefficient. Schools served as the units of randomization stratified by location (urban/rural) and by PE teacher status (certified/uncertified).	Even though results of the programme effect evaluation will be published elsewhere, preliminary analysis clearly indicates that the iPlay intervention resulted in a significant decrease in the incidence of PARI in the intervention group.
Mkumbo et al, 2009.[35]	Sexuality education in HIV/AIDS, STIs and unplanned pregnancy prevention.	Primary school students aged 12–14 years in Dar es Salaam, Tanzania. The sampling technique was not described.	Authors demonstrated good understanding and application of IM in developing a theory based sexuality education on HIV/AIDS, STIs and teenage pregnancy prevention intervention, describing the six steps.	Interviews, focus groups and quantitative surveys were conducted for needs assessment. Details of data collection and analysis for the intervention were not provided	Early sexual debut, multiple partners, and lack of condom use were found to be the main risky behaviours with the main determinants being: use of force by older men, gifts & favours and lack of knowledge and skills on condom use. Details of the programme effect evaluation is presented in a separate report cited in this article.
Wolfers et al, 2007.[25]	HIV/STIs prevention	Men with Afro-Caribbean and unmarried men with Turkish/Moroccan backgrounds in Rotterdam, the Netherlands.	Authors demonstrated good understanding and application of IM in developing a theory based HIV/STIs prevention intervention, but described only the first four steps.	Literature review, structured Interviews and focus groups were conducted for needs assessment. Details of data collection and analysis for the intervention were not provided	The determinants of prevention identified include: attitude, self-efficacy, socio-cultural factors, accessibility & availability of condoms and risk perceptions. Authors recommend a further research to evaluate programme effects.
Van Kesteren et al, 2006.[26]	Promotion of sexual health by preventing HIV/STI as well as ensuring a satisfactory sexual relationship, the Self-Help intervention	Dutch HIV-positive men who have sex with men in the Netherlands. Sampling technique was not described.	Authors demonstrated good understanding and application of IM in developing a theory based promotion of sexual health and HIV/STIs prevention intervention, describing the six steps.	Both qualitative and quantitative methods were used for needs assessments. HIV specialist nurses were used for programme implementation and data collection, but details and analysis were not provided.	Programme effect evaluation was planned to be presented in a separate paper.
Aaro et al, 2006.[18]	Prevention of HIV: Promotion of condom use and delaying onset sexual debut, the SATZ intervention.	Students aged 12–14 years in Dar es Salaam (Tanzania), Cape Town and Polokwane (South Africa). 24–30 schools (3000–5600 students) were selected from each study site and randomly allocated to intervention and control groups (cluster randomization).	Authors demonstrated good understanding and application of IM in developing a theory based promotion of condom use and delaying sexual debut, intervention, following the six steps	Data were collected using questionnaires at baseline, immediately after the intervention and after one year. Using a cluster effect 5.5% gave a power 80%, acceptable loss to follow up of 20% and required at least 11 pairs of schools. Therefore, 12, 13 and 15 pairs were respectively used for the three study sites.	Results of the evaluation were not provided
Fernandez et al, 2005.[33]	Breast and Cervical cancer screening using mammography and pap-smear test respectively, the “Cultivando La Salud” intervention	Hispanic farm-worker women aged 50 years and above in the United States of America. Sampling technique was not described	Authors demonstrated good understanding and application of IM in developing a theory based breast and cervical cancer screening intervention, following the six steps.	Literature review, focus groups, in-depth interviews and quantitative surveys were conducted for needs assessment. Details of data collection and analysis for the intervention were not provided	Determinants of screening were found to be physician referral, insurance coverage, access & regularity of care, cost, flexibility of place-of work policy, embarrassment & discomfort, fatalism, language barrier, fear of outcome & confidentiality, and lack of knowledge. A trial showed a 10.9% increase (29.9%-40.8%) in the uptake of mammography in the intervention group, and a 15.9% increase (23.6%-39.5%) the uptake of pap-smear test in the intervention group compared with the control group.
Hou et al, 2004.[34]	Pap-smear screening for cervical cancer, the “love yourself before you take care of your family” intervention.	Chinese women living in Taiwan. Sampling was not described. Sampling technique was not described	Authors demonstrated good understanding and application of IM in developing a theory base pap-smear screening intervention for cervical cancer, following the six steps	Focus groups and quantitative surveys were conducted for needs assessment. Details of data collection and analysis for the intervention were not provided.	Determinants of screening were found to be knowledge, perceived pros & cons to screening, and perceived norms about pap-smear screening. Intervention effect was to be evaluated in a separate RCT (Hou et al, 2002). However, preliminary results showed that women in the intervention group reported higher rate of completing the screening test than control (p = 0.002).
Van Empelen et al, 2003.[27]	Promotion of condom use to prevent HIV/AIDS.	Dutch drug users in the Netherlands. Sampling strategy was not described.	Authors demonstrated good understanding and application of IM in developing a theory base promotion of condom use intervention, following the six steps	Surveys and literature review were conducted for needs assessments. Details of data collection and analysis for the intervention were not provided.	Details of intervention evaluation and effect measures were not provided.
Hou et al, 2002.[23]	Individually randomized controlled trial of pap-smear screening for cervical cancer.	Chinese women aged 30 years and above (or younger if married), in Taiwan. Study population (424) was obtained from relatives of inpatient and randomly allocated to intervention and control groups.	Details of the IM framework application was described in different paper (Hou et al, 2004)	The primary outcome is screening behaviour (uptake) and intention in the following year assessed in a survey using pretested and evaluated instruments. Data was collected in both arms at baseline and after three months. Chi squared test was used to compare groups, while t-test and linear regression were used to analysed the mean scores of the secondary outcomes obtained on 5 point Likert scale.	51.2% of women in the intervention group and 31.5% in the control group reported having a pap-smear test within 3 months post intervention (p = 0.002). However, no significant difference in intention to take a pap-smear test between the two groups (p = 0.31).

**Table 4:** Summary of the critical appraisal.

Studies (Author, Date)	Clearly defined objectives	Possibility of selection bias	Appropriate study design	Identification & control of confounders	Any blinding	Appropriate data collection methods	Acceptable withdrawals and dropouts	Was the Intervention of acceptable integrity	Was data analysis clear & Robust	Strength of evidence
Byrd et al, 2013.[24]	Y	N	Y	N	N	Y	Y	Y	Y	Medium
Theunissen et al, 2013.[30]	Y	Y	Y	N	N	Y	O	Y	N	Weak
Riphagen-Dalhuisen et al, 2013.[21]	Y	N	Y	N	N	Y	O	Y	O	Weak
Byrd et al, 2012.[15]	Y	O	Y	N	N	Y	N	Y	N	Weak
Scarinci et al, 2012.[16]	O	O	Y	N	N	Y	N	Y	N	Weak
Wolfers et al, 2012.[28]	Y	O	Y	N	N	Y	N	Y	N	Weak
Van Der Veen et al, 2011.[29]	Y	O	Y	N	N	Y	N	Y	N	Weak
Looijmans-van den Akker et al, 2011.[31]	Y	N	Y	N	N	Y	N	Y	Y	Medium
Kok et al, 2011.[17]	Y	O	Y	N	N	N	N	Y	N	Weak
Collard et al, 2010.[20]	Y	N	Y	Y	N	Y	Y	Y	Y	Strong
Looijmans-van den Akker et al, 2010.[22]	Y	N	Y	N	N	Y	N	Y	Y	Strong
Corbie-Smith et al, 2010.[19]	Y	O	Y	N	N	N	N	Y	N	Weak
Schmid et al, 2010.[32]	Y	O	Y	N	N	Y	N	Y	N	Weak
Collard et al, 2009.[14]14	Y	N	Y	Y	N	Y	Y	Y	Y	Medium
Mkumbo et al, 2009.[35]	Y	O	Y	N	N	Y	N	Y	N	Weak
Wolfers et al, 2007.[25]	Y	O	Y	N	N	N	N	Y	N	Weak
Van Kesteren et al, 2006.[26]	Y	O	Y	N	N	O	N	Y	N	Weak
Aaro et al, 2006.[18]	Y	N	Y	Y	N	Y	Y	Y	Y	Medium
Fernández et al, 2005.[33]	Y	O	Y	N	N	Y	N	Y	N	Weak
Hou et al, 2004.[34]	Y	O	Y	Y	N	Y	N	Y	N	Weak
Van Empelen et al, 2003.[27]	Y	O	Y	N	N	Y	N	Y	N	Weak
Hou et al, 2002.[23]	Y	Y	Y	Y	N	Y	Y	Y	Y	Strong

Y = Yes, N = No, O = Not clear

### Summary of findings

Only five of the twenty two included studies provided the effects in terms of percentage increase in the uptake of prevention programmes, and only one of the five studies provided an effect measure (between interventions using IM protocol and interventions using placebo with standard care) in the form of relative risk (RR = 1.59, 95%CI = 1.08–2.34, p = 0.02). All the five studies were RCTs, three of which were rated as strong evidences, one as a medium evidence and one as a weak evidence. These studies are: 9% increase in the uptake of influenza vaccine (RR = 1.59, 95%CI = 1.08–2.34, p = 0.02),[22] 10.9% increase in the uptake of mammography & a 15.9% increase in the uptake of pap-smear test,[33] 19.7% increase in the uptake of pap-smear test (p = 0.002),[23] 28.5% increase in the uptake of pap-smear screening test (p<0.0001),[24] and a 50% reduction in physical activity-related injury among low active children (HR, 0.47; 95% CI, 0.21–1.06).[20] However, all the studies have identified the participants-tailored and theory-driven determinants of uptake of the respective disease prevention interventions, which is essential (or even a pre-requisite) to the success of any disease prevention programme. Results are presented by intervention/disease type:

#### HIV/STI prevention

Uptake of screening tests was found to be associated with attitude, self-efficacy, perceived norms of partners/friends/parents, perceived susceptibility, shame, pros, and characteristic test site accessibility. While the performance objectives for condom use were found to be; decide to use condom, obtain/buy condom, always carry condom along, be confident to negotiate using condom with your partner by communicating and persuading, agree to use condom or not to have sex, use condom correctly and persist on using condom for every act of sexual intercourse.[18,19,25–30,35]

#### Influenza prevention

The most important determinants of influenza vaccine uptake among HCWs were found to be: longer opening hours, more test locations, use of mobile carts, written policy, active request, working for more than 15 years, perceived personal risks, perceived reduction of risk to patients, awareness of the existence of guidelines and the influence of media attention on avian influenza.[17,21,31]

#### Cervical and breast cancer prevention

The determinants of screening uptake were found to be; knowledge, perception of susceptibility, perceived pros and cons, cultural norms, physician referral, insurance coverage, access & regularity of care, cost, flexibility of place-of work policy, embarrassment & discomfort, fatalism, language barrier and fear of outcome & confidentiality.[15,16,33,34]

#### Secondary stroke prevention

Determinants of prevention were found to be; the need for provider (discharge) check-off list, clinical reminders, training and education on risk factors & local resources, stroke support groups, IEC materials and administration support.[32]

#### Physical activity-related injury prevention

Determinants were found to be; type of sport played (contact/no contact), weather, time of season/time of day, playing surface, equipment (protective/footwear), rules, previous injury, age, sex, fitness level and psycho-social factors.[14]

## Discussion

Intervention mapping is now widely being used to design disease prevention interventions worldwide. It has been applied in a wide range of health promotion programmes including communicable and non-communicable disease preventions, as well as general health promotion. Most studies on intervention mapping reported that it has been found to be useful in designing disease prevention programmes, with varying degrees across different disease categories and populations. While some of the variations could be explained by differences in study designs and study populations, they also portray the need for methodological improvements in the use of intervention mapping to design prevention programmes.

Even though a meta-analysis could not be performed because only one study reported an effect estimate with confidence interval, an attempt is made to summarise the findings of the reviewed literature. All the five randomised controlled studies reported statistically significant difference between the IM intervention and placebo control groups, with the IM group associated with an increase in the uptake of disease-prevention intervention ranging from 9% to 28.5% (0.0001≤p≤0.02), and one study reported a 50% decrease in the incidence of physical activity-related injury among low active children (HR, 0.47; 95% CI, 0.21–1.06) in the IM group. On the other hand, all the 22 studies have successfully identified the determinants of the uptake of disease prevention interventions, which is essential to the success of disease prevention programmes.

The findings of this review show that uptakes of influenza vaccine among healthcare workers, mammography for breast cancer screening and pap-smear test for cervical cancer screening among sexually active women, as well as reduction in physical activity related injury among low active school children can be improved by designing disease prevention programmes using the intervention mapping protocol.[20,22–24,33] Since most of the identified determinants of the uptake of these prevention programmes are potentially modifiable, health planners can target to encourage (or discourage) their uptake through all the known effective ways, such as education, training and even incentives. This can be said with some level of certainty for pap-smear screening for breast cancer because three of the reviewed studies conducted in different populations are significantly associated with increased uptake. In the case influenza vaccine uptake, mammography for breast cancer and prevention of physical activity related injuries that were reported by only one study each, more work needs to be done to increase reliability of the findings. In the study on the prevention of physical activity related injury, the overall finding shows no statistically significant difference between the intervention (IM) and control groups, but a sub group analysis showed a 50% reduction in the incidence of injuries among the low active students in the intervention group. This shows that the outcome of interventions can be influenced by some specific characteristics of the study population, hence the need to design participants’ tailored interventions with detailed sub-group analyses.

Restricting the literature search to only English language published literature was a limitation, as this may limit the inclusion of useful evidence, thus possibly introducing some form of selection bias which makes generalization difficult. The study was also limited by the fact that 15 of the 22 studies reviewed were rated as weak evidences and 4 as medium evidences, thus making the evidence less reliable. The possibility of publication bias resulting in over-representation of the positive effects of interventions could not be ruled out, because studies with positive effects are more likely to be published and vice versa. However, this could not be assessed because only one study provided an effect estimate.

### Implications for practice, future research and policy

Even though the review process has some limitations, and the methodological qualities of most of the reviewed literature was also low, recommendations can be made to improve the design and implementation of intervention mapping on disease prevention programmes:

Most of the studies only described the development of disease prevention interventions using intervention mapping, but did not provide details of the epidemiological processes such as sampling techniques, methods of data collection and analysis, study design etc. Therefore, future studies on intervention mapping should take these into account in order to improve the methodological quality and validity of the studies. As much as possible, quantitative outcomes and effect estimates should be provided and where they are published in different articles, titles should refer to the previous articles. This is because the two articles obtained by contacting authors did not contain the key search term ‘intervention mapping’ in their titles and abstracts, hence, not captured by the search. Subsequent reviews on the effects of intervention mapping on disease prevention should, at the onset, focus on evaluation trials of interventions developed using intervention mapping (because they provide the effects needed) not on studies that just described how intervention mapping is used to develop a disease prevention intervention. However, there may be need to contact authors of the latter to get information on the former. There is need for larger reviews that would include all the relevant literature, which should be conducted in a more ideal setting and following all the principles and guidelines for conducting a systematic review. This will be needed to make some policy recommendations. There is a need to create global awareness and training on the use of intervention mapping in disease prevention, so that more research would be conducted in different parts of the world, which would add to the existing database. This is because most of the published studies were conducted in Europe and USA.

## Conclusion

Despite the widespread use of intervention mapping in designing disease prevention interventions, little evidence exist on magnitude of the role IM plays in promoting uptake of disease prevention interventions. IM has been successfully used to plan, implement and evaluate interventions that showed significant increase in uptake of disease prevention programmes. This study has found that disease prevention interventions that have used the intervention mapping approach have generally reported significant increases in the uptake of disease prevention programs.

This implies that it can be recommended for designing such interventions with some level of certainty. However, these findings should be interpreted with caution in making generalization because of the limitations of this review. Nonetheless, this has provided an insight on the role of intervention mapping in designing disease prevention interventions, and a good foundation upon which subsequent reviews can be planned and conducted. It is also recommended that the use of IM to promote primordial and secondary prevention should be reviewed.

## Supporting information

S1 Supporting Information (DOCX)
