# Supplementary material for: The role of intervention mapping in designing disease prevention interventions: A systematic review of the literature
Source: PLoS One. 2017 Mar 30;12(3):e0174438. doi: 10.1371/journal.pone.0174438 (PMC5373531; doi:10.1371/journal.pone.0174438)
Supplement: S1 Supporting Information — (DOCX) [file pone.0174438.s001.docx]

**File A. The PRISMA checklist**

| **Section/Topic** | **Item #** | **Checklist item** | **Reported on page #** |
| --- | --- | --- | --- |
| **TITLE** |  |  |  |
| Title | 1 | Identify the report as a systematic review, meta-analysis, or both | Cover |
| **ABSTRACT** |  |  |  |
| Structured summary | 2 | Provide a structured summary including, as applicable: background; objectives; data sources; study eligibility criteria, participants, and interventions; study appraisal and synthesis methods; results; limitations; conclusions and implications of key findings; systematic review registration number | 3 |
| **INTRODUCTION** | 3 |  |  |
| Rationale |  | Describe the rationale for the review in the context of what is already known | 8 |
| Objectives | 4 | Provide an explicit statement of questions being addressed with reference to participants, interventions, comparisons, outcomes, and study design (PICOS), | 8 |
| **METHODS** |  |  |  |
| Protocol and registration | 5 | Indicate if a review protocol exists, if and where it can be accessed (e.g. Web address), and, if available, provide registration information including registration number. | 8 |
| Eligibility criteria | 6 | Specify study characteristics (e.g., PICOS, length of follow-up) and report characteristics (e.g., years considered, language, publication status) used as criteria for eligibility, giving rationale. | 9 |
| Information sources | 7 | Describe all information sources (e.g., databases with dates of coverage, contact with study authors to identify additional studies) in the search and date last searched. | 9 |
| Search | 8 | Present full electronic search strategy for at least one database, including any limits used, such that it could be repeated. | 10 |
| Study selection | 9 | State the process for selecting studies (i.e., screening, eligibility, included in systematic review, and, if applicable, included in the meta-analysis). |  |
| Data collection process | 10 | Describe method of data extraction from reports (e.g., piloted forms, independently, in duplicate) and any processes for obtaining and confirming data from investigators. | 10 |
| Data items | 11 | List and define all variables for which data were sought (e.g., PiCOS, funding sources) and any assumptions and simplifications made. | 11 |
| Risk of bias in individual studies | 12 | Describe methods used for assessing risk of bias of individual studies (Including specification of whether this was done at the study or outcome level), and how this information is to be used in any data synthesis. | 11 |
| Summary measures | 13 | State the principal summary measures (e.g., risk ratio, difference in means | 11 |
| Synthesis of results | 14 | Describe the methods of handling data and combining results of studies, if done, including measures of consistency (e.g., 1^) for each meta-analysis |  |
| Risk of bias across studies | 15 | Specify any assessment of risk of bias that may affect the cumulative evidence (e.g., publication bias, selective reporting within studies). |  |
| Additional analysis | 16 | Describe methods of additional analyses (e.g., sensitivity or subgroup analyses, meta-regression), if done, indicating which were pre-specified. |  |
| **RESULTS** |  |  |  |
| Study selection | 17 | Give numbers of studies screened, assessed for eligibility, and included in the review, with reasons for exclusions at each stage, ideally with a flow diagram. | 11 |
| Study characteristics | 18 | For each study, present characteristics for which data were extracted (e.g., study size, PICOS, follow-up period) and provide the citations. | 12 |
| Risk of bias within studies | 19 | Present data on risk of bias of each study and, if available, any outcome-level assessment (see  Item 12), | 13 |

**The PRISMA checklist (continued)**

| **Section/Topic** | **Item #** | **Checklist item** | **Reported on page #** |
| --- | --- | --- | --- |
| Results of individual studies | 20 | For all outcomes considered (benefits or harms), present, for each study: (a) simple summary data for each intervention group and (b) effect estimates and confidence intervals, ideally with a forest plot. | 14 |
| Synthesis of results | 21 | Present results of each meta-analysis done, including confidence intervals and measures of consistency. |  |
| Risk of bias across studies. | 22 | Present results of any assessment of risk of bias across studies (see Item 15). | 13 |
| Additional analysis | 23 | Give results of additional analyses, if done (e.g., sensitivity or subgroup analyses, meta-regression  [see Item 16]). | 26 |
| **DISCUSSION** |  |  |  |
| Summary of evidence | 24 | Summarize the main findings including the strength of evidence for each main outcome; consider their relevance to key groups (e.g., health care providers, users, and policy makers). | 27 |
| Limitations | 25 | Discuss limitations at study and outcome level (e.g., risk of bias), and at review level (e.g., incomplete retrieval of identified research, reporting bias). | 28 |
| Conclusion | 26 | Provide a general interpretation of the results in the context of other evidence, and implications for future research. | 29 |
| **FUNDING** |  |  |  |
| Funding | 27 | Describe sources of funding for the systematic review and other support (e.g., supply of data); role of funders for the systematic review. |  |

**File B.** **Databases search**

| S/No | Search terms | Results |
| --- | --- | --- |
|  |  | MEDLINE: 0 |
| 1 | exp Intervention mapping/ | EMBASE: 0 |
|  |  | Web of Science: 0 |
|  |  | MEDLINE: 130 |
| 2 | Intervention mapping | EMBASE: 159 |
|  |  | Web of Science: 186 |
|  |  | MEDLINE: 130 |
| 3 | 1 OR 2 | EMBASE: 159 |
|  |  | Web of Science: 186 |
|  |  | MEDLINE: 9520233 |
| 4 | (HIV OR human immunodeficiency virus OR AIDS OR acquired immune deficiency syndrome OR hepatitis B virus OR HBV OR human papilloma virus OR HPV OR chlamydia OR influenza OR infect* OR injur* OR breast cancer OR cervical cancer OR prostate cancer OR colon cancer OR cancer OR drug* OR sex* OR smoking OR cigarette OR alcohol OR drinking OR binge OR wine OR bear OR behavi#r OR physical activity OR exercise sedentary OR inactivity OR psychiatr* OR psycholog* OR mental OR fruit OR vegetable OR diet OR nutrition* OR eat* OR feed* OR low calorie* OR low energy OR low fat OR low salt OR obes* OR weight gain OR overweight OR worker* OR student* OR depress* OR dementia OR stress OR chronic disease OR diabetes OR hypertension OR vaccin* OR stroke OR disabilit* OR asthma OR gynecolog* OR health promotion OR health education OR prevention OR ehealth OR family-based OR family OR school-based OR school OR web-based OR web OR workplace OR work related). | EMBASE: 15938151 |
|  |  | Web of Science: 12613343 |
|  |  | MEDLINE: 126 |
| 5 | 3 AND 4 | EMBASE: 154 |
|  |  | Web of Science: 178 |
|  |  |  |

**File C. Critical appraisal forms**

The Effective Public Health Practice Project (EPHPP) quality assessment tool for quantitative studies.

**Byrd et al, 2013**

| **S/No** | **Appraisal item** | **Comment** |
| --- | --- | --- |
| 1. | Was there any possibility of selection bias? | Somewhat likely, but randomization would have would have evenly distributed the possibility among the intervention and control groups |
| 2. | Was the research design appropriate to meet the research objectives? | Yes, individual RCT was appropriate for the programme evaluation. |
| 3. | Have confounders been identified and controlled? | Not stated, but randomization would have evenly distributed the effects of known and unknown confounders across the two study groups |
| 4. | Were participants and/or researchers blinded? | Not mentioned, but is not feasible to blind the participants. Analyses can be blinded |
| 5. | Was the data collection method appropriate for the research? | Yes, in-person recruitment alone is not appropriate, but validation from medical records makes it valid |
| 6. | Were withdrawal/ dropout rates acceptable? | Yes, only 100 participants (15.8%) were lost to follow up |
| 7. | Was the intervention of acceptable integrity? | Yes, the pap-smear screening intervention model was developed based on the intervention mapping protocol which is theory-based and participants tailored. |
| 8. | Was data analysis clear and robust? | Yes, as both intent-to-treat and per-protocol analyses were conducted and level of significance was also tested |

**Critical appraisal (continued)**

The Effective Public Health Practice Project (EPHPP) quality assessment tool for quantitative studies.

**Theunissen et al, 2013**

| **S/No** | **Appraisal item** | **Comment** |
| --- | --- | --- |
| 1. | Was there any possibility of selection bias? | Very likely, because sampling was based on web respondent driven chain referral and participants may differ significantly from non-participants |
| 2. | Was the research design appropriate to meet the research objectives? | Yes, because use of IM provided evidence-based intervention. And the web-based chain referral has proven good for sensitive issues like sexual activity |
| 3. | Have confounders been identified and controlled? | Not stated |
| 4. | Were participants and/or researchers blinded? | Not mentioned, but it does not appear feasible due to the nature of the intervention |
| 5. | Was the data collection method appropriate for the research? | Yes, structured interviews were used for need assessment and web-based questionnaires for the participants |
| 6. | Were withdrawal/ dropout rates acceptable? | Not feasible to obtain because sample size was not determined at the start of the study. It depends on the number of partners identified at the end |
| 7. | Was the intervention of acceptable integrity? | Yes, screening for chlamydia trachomatis is the most effective means of controlling and preventing the disease |
| 8. | Was data analysis clear and robust? | Not described in this paper. |

**Critical appraisal (continued)**

The Effective Public Health Practice Project (EPHPP) quality assessment tool for quantitative studies.

**Riphagen-Dalhuisen et al, 2013**

| **S/No** | **Appraisal item** | **Comment** |
| --- | --- | --- |
| 1. | Was there any possibility of selection bias? | Not likely, because it was a cluster randomized trial with all eligible members of the clusters enrolled in the study |
| 2. | Was the research design appropriate to meet the research objectives? | Yes, because IM provided an evidence based intervention strategy and the cluster randomization was minimized bias and confounding |
| 3. | Have confounders been identified and controlled? | No, but randomization would have balanced the effects of both known and unknown confounders. |
| 4. | Were participants and/or researchers blinded? | Not mentioned, but is not feasible to bind due to the nature of the intervention, except at the level of data analysis. |
| 5. | Was the data collection method appropriate for the research? | Yes, survey questionnaires for needs assessment and web-based questionnaires and interviews for evaluation were used for data collection at different stages. |
| 6. | Were withdrawal/ dropout rates acceptable? | Not stated for study implementation, but response rate was too low for the evaluation component (30.1% in the first season in 2010 and 18.6% in the second season in 2011). |
| 7. | Was the intervention of acceptable integrity? | Yes, vaccination of health care workers (HCWs) against influenza has been document to prevent both the HCWs and the patients under their care from developing the diseases. |
| 8. | Was data analysis clear and robust? | Not described in details |

**Critical appraisal (continued)**

The Effective Public Health Practice Project (EPHPP) quality assessment tool for quantitative studies.

**Byrd et al, 2012**

| **S/No** | **Appraisal item** | **Comment** |
| --- | --- | --- |
| 1. | Was there any possibility of selection bias? | Not likely, because systematic random sampling was used to select participants |
| 2. | Was the research design appropriate to meet the research objectives? | Yes, IM provided an evidence based intervention that was acceptable to the community members |
| 3. | Have confounders been identified and controlled? | Not mentioned |
| 4. | Were participants and/or researchers blinded? | Not mentioned, but does not appear feasible because of the nature of the intervention |
| 5. | Was the data collection method appropriate for the research? | Yes, focus groups and quantitative surveys were appropriate to the relevant stages of programme implementation |
| 6. | Were withdrawal/ dropout rates acceptable? | Not mentioned |
| 7. | Was the intervention of acceptable integrity? | Yes, Pap-smear screening is the known to be the most cost-effective preventive measure for cervical cancer. |
| 8. | Was data analysis clear and robust? | Not described in this paper. |

**Critical appraisal (continued)**

The Effective Public Health Practice Project (EPHPP) quality assessment tool for quantitative studies.

**Scarinci et al, 2012**

| **S/No** | **Appraisal item** | **Comment** |
| --- | --- | --- |
| 1. | Was there any possibility of selection bias? | Not clear, as the door to door method of sampling participants was not fully explained. |
| 2. | Was the research design appropriate to meet the research objectives? | Yes, IM provided an evidence-based intervention for both primary (sexual prevention) and secondary (pap smear) cervical cancer screening. |
| 3. | Have confounders been identified and controlled? | Not stated |
| 4. | Were participants and/or researchers blinded? | Not mentioned, but does not appear feasible due to the nature of the intervention, but researchers can be blinded during evaluation |
| 5. | Was the data collection method appropriate for the research? | Yes, the use of focus groups which guided the development of quantitative survey questionnaire was appropriate. |
| 6. | Were withdrawal/ dropout rates acceptable? | Not stated |
| 7. | Was the intervention of acceptable integrity? | Yes, both prevention of sexual transmission of human papilloma virus (HPV) and pap smear testing are very effective ways of preventing cervical cancer. |
| 8. | Was data analysis clear and robust? | Not described in this paper |

**Critical appraisal (continued)**

The Effective Public Health Practice Project (EPHPP) quality assessment tool for quantitative studies.

**Wolfers et al, 2012**

| **S/No** | **Appraisal item** | **Comment** |
| --- | --- | --- |
| 1. | Was there any possibility of selection bias? | Not clear, as sampling technique was not described in the article |
| 2. | Was the research design appropriate to meet the research objectives? | Yes, intervention mapping provided an evidence-based intervention for testing and prevention of sexually transmitted infections. |
| 3. | Have confounders been identified and controlled? | Not stated |
| 4. | Were participants and/or researchers blinded? | Not stated, but does not appear feasible due to the nature of the intervention, but researchers can be blinded during evaluation |
| 5. | Was the data collection method appropriate for the research? | Yes, the use of focus groups which guided the development of quantitative survey questionnaire was appropriate |
| 6. | Were withdrawal/ dropout rates acceptable? | Not stated |
| 7. | Was the intervention of acceptable integrity? | Yes, testing for sexually transmitted infections is effective in the prevention of all the respective infections. |
| 8. | Was data analysis clear and robust? | Not described in this paper |

**Critical appraisal (continued)**

The Effective Public Health Practice Project (EPHPP) quality assessment tool for quantitative studies.

**Van Der Veen et al, 2011**

| **S/No** | **Appraisal item** | **Comment** |
| --- | --- | --- |
| 1. | Was there any possibility of selection bias? | Not clear as the sampling technique was not explained in this paper |
| 2. | Was the research design appropriate to meet the research objectives? | Yes, Intervention mapping provided an evidence based intervention |
| 3. | Have confounders been identified and controlled? | Not stated in this paper |
| 4. | Were participants and/or researchers blinded? | Not stated, but does not appear feasible at the level of implementation of the intervention, except during evaluation. |
| 5. | Was the data collection method appropriate for the research? | Yes, because 86% of the participants reported having access to internet at home daily, while the remaining were contacted through phones. |
| 6. | Were withdrawal/ dropout rates acceptable? | Not stated |
| 7. | Was the intervention of acceptable integrity? | Yes, HBV screening is known to be one of the most effective ways of preventing the disease through early diagnosis and treatment. |
| 8. | Was data analysis clear and robust? | Not described in this paper, described in a separate randomized controlled trial |

**Critical appraisal (continued)**

The Effective Public Health Practice Project (EPHPP) quality assessment tool for quantitative studies.

**Looijmans-van den Akker et al, 2011**

| **S/No** | **Appraisal item** | **Comment** |
| --- | --- | --- |
| 1. | Was there any possibility of selection bias? | Somewhat likely, as participants’ selection was said to be based on random sampling, although not described. |
| 2. | Was the research design appropriate to meet the research objectives? | Yes, , intervention mapping provided an evidence based intervention and cluster randomized control trial was also appropriate for measuring the effect estimate |
| 3. | Have confounders been identified and controlled? | Not stated |
| 4. | Were participants and/or researchers blinded? | Not mentioned, but does appear feasible due to the nature of the intervention, except at the level of evaluation which is described in separate paper. |
| 5. | Was the data collection method appropriate for the research? | Yes, in-depth interviews, focus groups and quantitative surveys were used appropriate at the different stages of the study |
| 6. | Were withdrawal/ dropout rates acceptable? | Not stated, however, response rate for the needs assessment survey was low, 45% |
| 7. | Was the intervention of acceptable integrity? | Yes, a Cochrane review showed that influenza vaccination of health workers reduces all-cause mortality by 32% (95% CI: 16 to 45%) |
| 8. | Was data analysis clear and robust? | Only the needs assessment data was described in details but the evaluation RCT was described in a separate paper. |

**Critical appraisal (continued)**

The Effective Public Health Practice Project (EPHPP) quality assessment tool for quantitative studies.

**Kok et al, 2011**

| **S/No** | **Appraisal item** | **Comment** |
| --- | --- | --- |
| 1. | Was there any possibility of selection bias? | Not clear, sampling technique not mentioned |
| 2. | Was the research design appropriate to meet the research objectives? | Yes, intervention mapping provided an evidence based intervention |
| 3. | Have confounders been identified and controlled? | Not stated |
| 4. | Were participants and/or researchers blinded? | Not mentioned, but does not appear feasible at the level of implementation of the intervention, except during evaluation |
| 5. | Was the data collection method appropriate for the research? | Not described |
| 6. | Were withdrawal/ dropout rates acceptable? | Not stated |
| 7. | Was the intervention of acceptable integrity? | Yes, vaccination against influenza is known to be the best preventive measure. |
| 8. | Was data analysis clear and robust? | Not described |

**Critical appraisal (continued)**

The Effective Public Health Practice Project (EPHPP) quality assessment tool for quantitative studies.

**Collard et al, 2010**

| **S/No** | **Appraisal item** | **Comment** |
| --- | --- | --- |
| 1. | Was there any possibility of selection bias? | Very unlikely, because schools were randomly selected and randomized as clusters |
| 2. | Was the research design appropriate to meet the research objectives? | Yes, cluster RCT was appropriate for the effect evaluation of the intervention |
| 3. | Have confounders been identified and controlled? | Yes, data analysis was stratified by potential confounders; age, sex, geographical location and qualification of PE teachers. Effect modifiers were also assessed; BMI, ethnicity, urbanization, PA exposure time etc. |
| 4. | Were participants and/or researchers blinded? | Not stated, but it would be difficult to blind participants. |
| 5. | Was the data collection method appropriate for the research? | Yes, recording injuries by trained physical education teachers was appropriate |
| 6. | Were withdrawal/ dropout rates acceptable? | Yes, drop out rates were 8.9% and 8.2% in the intervention and control groups respectively |
| 7. | Was the intervention of acceptable integrity? | Yes, the iPlay intervention was developed based on the intervention mapping protocol which is theory based and participants tailored. |
| 8. | Was data analysis clear and robust? | Yes, intention to treat analysis was performed different regression models were used. Results were presented in tables and charts. |

**Critical appraisal (continued)**

The Effective Public Health Practice Project (EPHPP) quality assessment tool for quantitative studies.

**Looijmans-van den Akker et al, 2010**

| **S/No** | **Appraisal item** | **Comment** |
| --- | --- | --- |
| 1. | Was there any possibility of selection bias? | Very likely, because only 11% of the nursing homes agreed to participate in the study and they could have differed significantly from those that refused to participate |
| 2. | Was the research design appropriate to meet the research objectives? | Yes, cluster RCT was appropriate for the research. |
| 3. | Have confounders been identified and controlled? | Not stated, but randomization would have equally distribute the effects of known and unknown confounders across the two groups |
| 4. | Were participants and/or researchers blinded? | Not stated, but does not appear feasible due to the nature of the intervention, but data analysis could be blinded even though not mentioned. |
| 5. | Was the data collection method appropriate for the research? | Yes, obtaining data from vaccination card was appropriate. |
| 6. | Were withdrawal/ dropout rates acceptable? | Not stated |
| 7. | Was the intervention of acceptable integrity? | Yes, a Cochrane review showed that influenza vaccination of health workers reduces all-cause mortality by 32% (95% CI: 16 to 45%) |
| 8. | Was data analysis clear and robust? | Yes, detailed and robust analysis was presented in numbers and tables. |

**Critical appraisal (continued)**

The Effective Public Health Practice Project (EPHPP) quality assessment tool for quantitative studies.

**Corbie-Smith et al, 2010**

| **S/No** | **Appraisal item** | **Comment** |
| --- | --- | --- |
| 1. | Was there any possibility of selection bias? | Not clear, as sampling technique was not described |
| 2. | Was the research design appropriate to meet the research objectives? | Yes, intervention mapping provided an evidence based intervention |
| 3. | Have confounders been identified and controlled? | Not stated |
| 4. | Were participants and/or researchers blinded? | Not stated, but does not appear feasible due to the nature of the intervention |
| 5. | Was the data collection method appropriate for the research? | Not described |
| 6. | Were withdrawal/ dropout rates acceptable? | Not stated |
| 7. | Was the intervention of acceptable integrity? | Yes, using the lay health advisor model has been found to be effective and acceptable for sexuality issues interventions. |
| 8. | Was data analysis clear and robust? | Not described |

**Critical appraisal (continued)**

The Effective Public Health Practice Project (EPHPP) quality assessment tool for quantitative studies.

**Schmid et al, 2010**

| **S/No** | **Appraisal item** | **Comment** |
| --- | --- | --- |
| 1. | Was there any possibility of selection bias? | Not clear, sampling technique was not described |
| 2. | Was the research design appropriate to meet the research objectives? | Yes, intervention mapping provided an evidence based intervention |
| 3. | Have confounders been identified and controlled? | Not stated |
| 4. | Were participants and/or researchers blinded? | Not mentioned |
| 5. | Was the data collection method appropriate for the research? | Data collection method for the needs assessment was appropriate, but that of the intervention was not described |
| 6. | Were withdrawal/ dropout rates acceptable? | Not stated |
| 7. | Was the intervention of acceptable integrity? | Yes, locally tailored prevention intervention using the available local resources was appropriate |
| 8. | Was data analysis clear and robust? | Only analysis of the needs assessment interviews was described which was robust. But analysis of the programme evaluation was not described. |

**Critical appraisal (continued)**

The Effective Public Health Practice Project (EPHPP) quality assessment tool for quantitative studies.

**Collard et al, 2009**

| **S/No** | **Appraisal item** | **Comment** |
| --- | --- | --- |
| 1. | Was there any possibility of selection bias? | Not likely, because of random selection of clusters and randomization of selected clusters into intervention and control groups |
| 2. | Was the research design appropriate to meet the research objectives? | Yes, intervention mapping provided an evidence based intervention and cluster randomization was appropriate for evaluating the intervention |
| 3. | Have confounders been identified and controlled? | Yes, this led to stratification of clusters by location (urban/rural) and by PE teacher status (certified/uncertified). Also randomization would take care of unknown confounders. |
| 4. | Were participants and/or researchers blinded? | Not mentioned, but does not appear feasible, except at the level of data analysis |
| 5. | Was the data collection method appropriate for the research? | Yes, use of PE teachers to identify injured children to fill a questionnaire was considered appropriate for the study. |
| 6. | Were withdrawal/ dropout rates acceptable? | Yes, considering a dropout rate of 20% gave a minimum sample size of 2280, which was applied. |
| 7. | Was the intervention of acceptable integrity? | Yes, because it was designed based on findings of the stakeholders-based needs assessment, and it was pretested |
| 8. | Was data analysis clear and robust? | Yes, data analysis was described in a very clear and systemic way, including plan to use a multiple regression analysis for the effect evaluation. |

**Critical appraisal (continued)**

The Effective Public Health Practice Project (EPHPP) quality assessment tool for quantitative studies.

**Mkumbo et al, 2009**

| **S/No** | **Appraisal item** | **Comment** |
| --- | --- | --- |
| 1. | Was there any possibility of selection bias? | Not clear, as the intervention sampling technique was not described. |
| 2. | Was the research design appropriate to meet the research objectives? | Yes, intervention mapping has been used to provide evidence based HIV/STI prevention interventions. |
| 3. | Have confounders been identified and controlled? | Not stated |
| 4. | Were participants and/or researchers blinded? | Not mentioned, but does not appear feasible due to the nature of the intervention |
| 5. | Was the data collection method appropriate for the research? | Only data collection for needs assessments was described which was appropriate for the intervention type and study population |
| 6. | Were withdrawal/ dropout rates acceptable? | Not stated |
| 7. | Was the intervention of acceptable integrity? | Yes, sexuality education is a documented way of preventing HIV/AIDS, STI and teenage pregnancy |
| 8. | Was data analysis clear and robust? | Not provided. Said to be described in a separate paper. |

**Critical appraisal (continued)**

The Effective Public Health Practice Project (EPHPP) quality assessment tool for quantitative studies.

**Wolfers et al, 2007**

| **S/No** | **Appraisal item** | **Comment** |
| --- | --- | --- |
| 1. | Was there any possibility of selection bias? | Not clear, as the intervention sampling technique was not described. |
| 2. | Was the research design appropriate to meet the research objectives? | Yes, intervention mapping has been used to provide evidence based HIV/STI prevention interventions. |
| 3. | Have confounders been identified and controlled? | Not stated |
| 4. | Were participants and/or researchers blinded? | Not stated, but does not appear feasible due to the nature of the intervention |
| 5. | Was the data collection method appropriate for the research? | Needs assessments data collection was appropriate for the intervention type and study population, but that of the intervention was not described. |
| 6. | Were withdrawal/ dropout rates acceptable? | Not stated |
| 7. | Was the intervention of acceptable integrity? | Yes, use of modelling, posters, play cards and provision of basic factual information are known to be effective in sexuality issues. |
| 8. | Was data analysis clear and robust? | Not provided, and no plan for evaluation |

**Critical appraisal (continued)**

The Effective Public Health Practice Project (EPHPP) quality assessment tool for quantitative studies.

**Van Kesteren et al, 2006**

| **S/No** | **Appraisal item** | **Comment** |
| --- | --- | --- |
| 1. | Was there any possibility of selection bias? | Not clear, as the sampling technique was not described. |
| 2. | Was the research design appropriate to meet the research objectives? | Yes, intervention mapping has been used to provide evidence based sexual health promotion and HIV/STI prevention interventions. |
| 3. | Have confounders been identified and controlled? | Not stated |
| 4. | Were participants and/or researchers blinded? | Not feasible due to the nature of the intervention |
| 5. | Was the data collection method appropriate for the research? | Not described |
| 6. | Were withdrawal/ dropout rates acceptable? | Not stated |
| 7. | Was the intervention of acceptable integrity? | Yes, use of behaviour change plan, consciousness raising, dramatic relief, environmental re-evaluation, systematic desensitization and anticipated regret was valid in sexuality research. |
| 8. | Was data analysis clear and robust? | Intervention evaluation and analysis was planned to be presented in a separate paper. |

**Critical appraisal (continued)**

The Effective Public Health Practice Project (EPHPP) quality assessment tool for quantitative studies.

**Aaro et al, 2006**

| **S/No** | **Appraisal item** | **Comment** |
| --- | --- | --- |
| 1. | Was there any possibility of selection bias? | Not likely due to cluster randomization |
| 2. | Was the research design appropriate to meet the research objectives? | Yes, cluster randomization using IM in the intervention group was appropriate |
| 3. | Have confounders been identified and controlled? | Yes, by stratification and randomization |
| 4. | Were participants and/or researchers blinded? | Not mentioned, but it was not feasible to blind the participants |
| 5. | Was the data collection method appropriate for the research? | Yes, use of questionnaires translated into local languages was appropriate. |
| 6. | Were withdrawal/ dropout rates acceptable? | Yes, cluster sample size was large enough to take care of loss to follow up of 20% |
| 7. | Was the intervention of acceptable integrity? | Yes, promotion of condom use and delaying the onset of sexual debut using role playing and skills training was appropriate. |
| 8. | Was data analysis clear and robust? | Yes, very clear and robust analysis was described but results were not provided. |

**Critical appraisal (continued)**

The Effective Public Health Practice Project (EPHPP) quality assessment tool for quantitative studies.

**Fernandez et al, 2005**

| **S/No** | **Appraisal item** | **Comment** |
| --- | --- | --- |
| 1. | Was there any possibility of selection bias? | Not clear, as the sampling technique was not described. |
| 2. | Was the research design appropriate to meet the research objectives? | Yes, intervention mapping provided an evidence-based interventions |
| 3. | Have confounders been identified and controlled? | Not stated |
| 4. | Were participants and/or researchers blinded? | Not stated, but does not appear feasible due to the nature of the intervention |
| 5. | Was the data collection method appropriate for the research? | Not described |
| 6. | Were withdrawal/ dropout rates acceptable? | Not stated |
| 7. | Was the intervention of acceptable integrity? | Yes, mammography and pap-smear test are very for the prevention of breast and cervical cancers respectively. |
| 8. | Was data analysis clear and robust? | Not provided in this paper |

**Critical appraisal (continued)**

The Effective Public Health Practice Project (EPHPP) quality assessment tool for quantitative studies.

**Hou et al, 2004**

| **S/No** | **Appraisal item** | **Comment** |
| --- | --- | --- |
| 1. | Was there any possibility of selection bias? | Not clear, because the sampling technique was not described. |
| 2. | Was the research design appropriate to meet the research objectives? | Yes, intervention mapping provided an evidence-based pap-smear screening intervention. |
| 3. | Have confounders been identified and controlled? | Yes, socio-demographic and sub-groups differences were taken into account during programme design |
| 4. | Were participants and/or researchers blinded? | Not stated, but does not appear feasible due to the nature of the intervention |
| 5. | Was the data collection method appropriate for the research? | Only that of needs assessment was described and found to be appropriate. |
| 6. | Were withdrawal/ dropout rates acceptable? | Not stated |
| 7. | Was the intervention of acceptable integrity? | Yes, pap-smear test is very effective for the prevention cervical cancer. |
| 8. | Was data analysis clear and robust? | Not provided in this paper. |

**Critical appraisal (continued)**

The Effective Public Health Practice Project (EPHPP) quality assessment tool for quantitative studies.

**Van Empelen et al, 2003**

| **S/No** | **Appraisal item** | **Comment** |
| --- | --- | --- |
| 1. | Was there any possibility of selection bias? | Not clear, as the sampling technique was not described. |
| 2. | Was the research design appropriate to meet the research objectives? | Yes, intervention mapping provided an evidence-based promotion of condom use interventions |
| 3. | Have confounders been identified and controlled? | Not stated |
| 4. | Were participants and/or researchers blinded? | Not mentioned, but does not appear feasible due to the nature of the intervention |
| 5. | Was the data collection method appropriate for the research? | Not described. |
| 6. | Were withdrawal/ dropout rates acceptable? | Not stated |
| 7. | Was the intervention of acceptable integrity? | Yes, use of condom is one of the most effective ways of preventing HIV/AIDS and other STIs. |
| 8. | Was data analysis clear and robust? | Not provided in this paper |

**Critical appraisal (continued)**

The Effective Public Health Practice Project (EPHPP) quality assessment tool for quantitative studies.

Hou et al, 2002

| **S/No** | **Appraisal item** | **Comment** |
| --- | --- | --- |
| 1. | Was there any possibility of selection bias? | Somewhat likely, because the study population (relatives of inpatients) could be different from the general population |
| 2. | Was the research design appropriate to meet the research objectives? | Yes, individually randomized trial, using IM for the intervention was appropriate. |
| 3. | Have confounders been identified and controlled? | Not clearly stated, but both arms were said not to be significantly different at baseline, which could imply fairly equal distribution of confounders |
| 4. | Were participants and/or researchers blinded? | Not stated, but does not appear feasible (except at the level of analysis) due to the nature of the intervention. |
| 5. | Was the data collection method appropriate for the research? | Yes, use of pretested and evaluated questionnaire was appropriate. |
| 6. | Were withdrawal/ dropout rates acceptable? | Fair enough, as 65% of the eligible women agreed to participate in the study, with an overall response rate of 58%. |
| 7. | Was the intervention of acceptable integrity? | Yes, pap-smear screening is one of the most effective ways of preventing cervical cancer. |
| 8. | Was data analysis clear and robust? | Yes, there was clear definition of outcomes and the use of chi squared test, t-test, and regression models for comparism. |
